# Correlation of N-acetyltransferase 2 genotype and acetylation status with plasma isoniazid concentration and its metabolic ratio in ethiopian tuberculosis patients

**DOI:** 10.1038/s41598-023-38716-3

**Published:** 2023-07-15

**Authors:** Tesemma Sileshi, Nigus Fikrie Telele, Victoria Burkley, Eyasu Makonnen, Eleni Aklillu

**Affiliations:** 1 Department of Pharmacy, Ambo University, Ambo, Ethiopia; 2 Department of Pharmacology and Clinical Pharmacy, Addis Ababa University, Addis Ababa, Ethiopia; 3 Department of Laboratory Medicines, Karolinska Institutet, Stockholm, Sweden; 4 Center for Innovative Drug Development and Therapeutic Trials for Africa (CDT-Africa), Addis Ababa University, Addis Ababa, Ethiopia; 5 Department of Global Public Health, Karolinska Institutet, Stockholm, Sweden

**Keywords:** Microbiology, Medical research, Tuberculosis, Genetics, Pharmacogenomics

## Abstract

Unfavorable treatment outcomes for tuberculosis (TB) treatment might result from altered plasma exposure to antitubercular drugs in TB patients. The present study investigated the distribution of the N-Acetyltransferase 2 (*NAT2*) genotype, isoniazid acetylation status, genotype–phenotype concordance of NAT2, and isoniazid plasma exposure among Ethiopian tuberculosis patients. Blood samples were collected from newly diagnosed TB patients receiving a fixed dose combination of first-line antitubercular drugs daily. Genotyping of *NAT2* was done using TaqMan drug metabolism assay. Isoniazid and its metabolite concentration were determined using validated liquid chromatography-tandem mass spectrometry (LC–MS/MS). A total of 120 patients (63 male and 57 female) were enrolled in this study. The mean daily dose of isoniazid was 4.71 mg/kg. The frequency of slow, intermediate, and fast *NAT2* acetylators genotypes were 74.2%, 22.4%, and 3.3% respectively. The overall median isoniazid maximum plasma concentration (C_max_) was 4.77 µg/mL and the AUC_0–7 h_ was 11.21 µg.h/mL. The median C_max_ in slow, intermediate, and fast acetylators were 5.65, 3.44, and 2.47 μg/mL, respectively. The median AUC_0–7 h_ hour in slow, intermediate, and fast acetylators were 13.1, 6.086, and 3.73 mg•h/L, respectively. The majority (87.5%) of the study participants achieved isoniazid C_max_ of above 3 µg/mL, which is considered a lower limit for a favorable treatment outcome. There is 85% concordance between the *NAT2* genotype and acetylation phenotypes. *NAT2* genotype, female sex, and dose were independent predictors of C_max_ and AUC_0–7 h_ (*p* < 0.001). Our finding revealed that there is a high frequency of slow *NAT2* genotypes. The plasma C_max_ of isoniazid was higher in the female and slow acetylators genotype group. The overall target plasma isoniazid concentrations in Ethiopian tuberculosis patients were achieved in the majority of the patients. Therefore, it is important to monitor adverse drug reactions and the use of a higher dose of isoniazid should be closely monitored.

## Introduction

Tuberculosis (TB) remains the major cause of death from infectious diseases. About 1.6 million people died from TB. This makes TB the second leading killer infectious disease after COVID-19 in 2021^1^. Although the six-month treatments for tuberculosis with the first-line antitubercular drugs (rifampicin, isoniazid, pyrazinamide, ethambutol) are effective, the emergence of drug resistance (DR-TB) impacts TB control success achieved^2,3^. DR-TB emerges because of inadequate or interrupted drug use, and the infecting mycobacteria are partially drug-resistant^4^. An increased drug exposure improved treatment outcomes; showing a positive relationship between anti-tuberculosis drug exposure and treatment outcome^5,6^.

The introduction of isoniazid, which is relatively inexpensive, and well tolerated in 1952, for tuberculosis care opened the modern era of tuberculosis treatment^7^. Isoniazid has high early bactericidal activity (EBA) and it can reduce bacterial load by 90–95% in the first 2 days of treatment^8^. EBA activity of isoniazid depends on the concentration that reaches the bacilli^9^. Several pharmacokinetic studies suggest a target of 3–6 μg/mL for the peak concentration (C_max_) following a 300 mg once-daily dose of isoniazid. The C_max_ of isoniazid occurs 1–2 h post-dose^10^.

Isoniazid is primarily metabolized to N-acetyl- isoniazid by the arylamine N-acetyltransferase 2 (NAT2) enzyme^11^. The *NAT2* gene is located on chromosome 8p22 and encodes the NAT2 enzyme^12^. *NAT2* gene is highly polymorphic displaying wide between-patient and between-population variations in its expression and enzyme activity. NAT2*4 is a wild-type allele which is a fast acetylator genotype, while the common defective variant alleles (NAT2*5, *6, *7, and *14) result in decreased acetylation activity and slow acetylation status. Intermediate acetylators carry one copy of NAT2*4^13^. Identification of *NAT2* polymorphism is useful to predict the effective therapeutic doses and adverse effects of isoniazid in different acetylators groups^14,15^. Slow acetylators are at increased risk of toxicity from isoniazid^16,17^ while fast acetylators are at increased risk of treatment failure^6^.

Isoniazid is metabolized to N-acetylisoniazid (AcINH) by the NAT2 enzyme, isonicotinic acid (INA), and hydrazine (Hz) by the amidase enzyme. NAT2 also catalyzes the acetylation of acetyl hydrazine (AcHz), which is a metabolite of AcINH, to non-toxic diacetylhydrazine. It also undergoes non-enzymatic conjugation with various endogenous substrates such as vitamin B6^18^. The mechanism by which isoniazid induces liver injury is not well established but believed that the metabolism of isoniazid produces a reactive metabolite that causes liver damage. Nevertheless, several recent studies showed that slow acetylators are at increased risk of hepatotoxicity^14,16–18^ .

The distribution of slow acetylator and *NAT-2* defective variant allele frequency varies across regions and populations within the region^19,20^. Black Africans display wide variations in *NAT2* genotype frequencies and slow acetylator phenotypes than non-Africans. Similarly, previous studies reported a high frequency of slow acetylator genotypes and phenotypes in the Ethiopian population^21,22^. Higher plasma isoniazid concentration was observed in Ethiopian pediatric patients^21^. On another hand, sub-clinical hepatotoxicity was observed in 17.3% of the patients who received the first-line antitubercular drugs^23^. 

Ethiopia is listed among the top 20 high TB and TB/HIV burden countries globally^24^. Isoniazid is part of the first-line anti-TB regimen in the country. Variations in the isoniazid acetylation rate, partly due to *NAT2* genetic variation, may influence TB treatment outcomes. But data is lacking on the distribution of the NAT2 acetylation status and its relationship with plasma isoniazid concentrations among Ethiopian TB patients. Therefore, this study investigated the distribution of the *NAT2* genotype-based acetylation status and its correlation with the C_max_ and plasma exposure (AUC _0–7 h_) of isoniazid in Ethiopian tuberculosis patients.

## Materials and methods

### Study participants

The study population comprised adult TB patients aged 18–65 years, receiving standard first-line drugs for TB treatment according to the Ethiopian treatment guidelines^25^. Newly diagnosed patients with drug-susceptible *Mycobacterium* TB were recruited from the TB clinics of the health center found in Addis Ababa (Beletshachew, Teklehymanote, Kazanchis, Woreda 2, and Areda Health Centre) from October 2019 to November 2021. Patients with either pulmonary or extrapulmonary forms of TB were included in the study. Patients received a daily dose of fixed-dose combination tablets containing 150, 75, 400, and 275 mg of rifampicin, isoniazid, pyrazinamide, and ethambutol respectively. The number of tablets received daily was based on the patient's body weight. Patients with a body weight greater than 55 kg received four fixed-dose combinations (FDC) tablets daily. Patients with a body weight between 40 and 55 kg received three FDC tablets daily and those under 40 received two FDC tablets. Treatment was provided under directly observed therapy (DOTs) at a primary health care facility found in Addis Ababa.

The study received ethical approval from the Institutional Review Board of the College of Health Science, Addis Ababa University (Ref number 080/17/IM), and the national research ethics review committee (Ref. Number MoSHE/RD/401/10,975/20). The study was conducted following the ethical principle of the Helsinki Declaration. All participants received a detailed explanation of the study protocol and provided written informed consent.

### Blood sample collection

Blood samples were collected after observing drug intake in an EDTA tube. The sample was collected 2 weeks post-treatment initiation and only during the intensive phases of treatment. Blood samples were drawn at three-time points ranging from 1 to 7 h post-drug intake. But for a few patients, blood samples were drawn at two-time points. Plasma was separated immediately and stored at − 80 °C at the Department of Pharmacology and Clinical Pharmacy at Addis Ababa University until being transported to Karolinska Institutet in Stockholm, Sweden for analysis on dry ice.

### DNA extraction and SNP genotyping

Genomic DNA was extracted from whole blood samples using the QIAmp DNA Blood Midi Kit (QIAGEN GmbH, Hilden, Germany) following the manufacturer's protocol. DNA was quantified using a NanoDrop spectrophotometer (Thermo Scientific) and stored at –20 °C until genotyping assay analysis. The recommended 4-SNP genotype panel of NAT2*5 (c.341 T > C), NAT2*6 (c.590G > A), NAT2*7 (c.857G > A), NAT2*14 (191G > A, rs1801279) for reliable estimation of rapid, intermediate, and slow acetylator phenotypes were selected^13,26^. Genotyping was performed using TaqMan drug metabolism assay reagents for allelic discrimination (Applied Biosystems Genotyping Assays) with the following ID numbers for each SNP: C___1204093_20 for NAT2*5 (c.341 T > C, rs1801280), C___1204091_10 for NAT2*6 (c.590G > A, rs1799930), C____572770_20 for NAT2*7 (c.857G > A, rs1799931), C____572770_20 for NAT2*14 (191G > A, rs1801279). The final volume for each reaction was 10 μL, consisting of 9 μL TaqMan® fast advanced master mix (Applied Biosystems, Waltham, MA, United States), DNA/RNA free water, TaqMan 40X for all *NAT2*, drug metabolism genotyping assays mix (Applied Biosystems) and 1 μL genomic DNA.

Genotyping was performed by real-time Q-PCR (Applied Biosystems) equipped with 7500 software V2.3 (life technologies corporation) for allelic discrimination. The PCR conditions consisted of an initial step at 60 °C for 30 s, hold stage at 95 °C for 10 min and PCR stage for 40 cycles, step 1 at 95 °C for 15 min and step 2 at 60 °C for 1 min, and after reading stage with 60 °C for 30 s.

### Quantification of plasma isoniazid and its metabolite concentration

For the determination of plasma concentration of isoniazid 4 mL venous blood was collected 2 weeks post-treatment initiation in the morning after an overnight fast in an EDTA tube. Plasma was separated immediately and stored at − 80 °C at the department of pharmacology and clinical pharmacy, Addis Ababa University until transported to Karolinska Institutet, Stockholm, Sweden for analysis. Quantification of isoniazid and acetyl-isoniazid were done at the therapeutic drug monitoring laboratory, Department of Clinical Pharmacology, Karolinska University Hospital. In brief, the concentration of isoniazid and acetyl-isoniazid were determined simultaneously using a liquid chromatography-tandem mass spectrometry (LC–MS/MS) system consisting of an Acquity Ultra Performance LC-system coupled to a Xevo TQ-S Micro (Waters, Milford, MA, USA). The chromatographic column consisted of YMC-ultraHT hydrosphere C18, 2 μm, 100 × 2 mm, reversed-phase column (Waters). And the mobile phase gradient of 0.1% formic acid in Milli-Q pure water, 100% methanol: methanol/Milli-Q pure water: Formic acid (10:90:0.1), methanol: Milli-Q pure water: isopropanol: Formic acid (70: 20: 10: 0.1), Methanol: Milli-Q pure water (10:90). The plasma sample preparation was based on protein precipitation with acetonitrile containing Isoniazid-d4, and Acetylisoniazid-d4 as an internal standard. The lower limit of quantification for isoniazid and acetyl-isoniazid were 0.05 µg/mL and 0.05 µg/mL respectively and the quantification ranges were 0.05–20 µg/mL and 0.05–10 µg/mL respectively. The method was validated according to the European Medicines Agency Guideline on bioanalytical criteria^27^.

### Statistical analysis

For each patient, the C_max_ was defined as the highest concentration measured, and the Tmax was the time point at which the C_max_ occurred. AUC_0–7 h_ calculation was performed using the trapezoidal rule. Graphpad prism was used to calculate AUC_0-7 h_. Continuous data were presented as median (interquartile range) for non-normal distributed data and mean standard deviation for normally distributed data. The Chi-square test was used to assess the Hardy–Weinberg equilibrium and genotype–phenotype concordance. Kruskal–Wallis tests were performed to see differences in C_max_ of isoniazid and acetyl isoniazid concentrations among the different genotypes. Univariate followed by stepwise multivariate linear regression analysis was performed to identify a predictive factor of isoniazid C_max_ and AUC_0-7 h_. Statistical analyses were performed using SPSS, version 27. *P* value < 0.05 was considered statistically significant.

## Results

### Patient characteristics

A total of 120 newly diagnosed tuberculosis patients who were non-diabetic and HIV-negative (63 males and 57 females) were included in this study. The detailed patient characteristic is described in Table 1. Nearly two-thirds of the patients had pulmonary tuberculosis. The median age of the patients was 28 years (IQR, 22–35). The mean dose of isoniazid received was 4.7 mg/kg/day (4.6–4.78, 95% CI). The documented rate of substance use was 13.33%, 17.5%, and 16.67% for cigarettes, khat, and alcohol respectively. Overall, 96.7% (N = 116) of participants completed treatment; 2 (1.67%) were lost to follow-up, and 2 (1.67%) were transferred to another health facility. Only one patient showed treatment failure. None of the study participants discontinued treatment because of the medication's adverse effects.

**Table 1:** Study participants' sociodemographic characteristics stratified by type of tuberculosis infection (n = 120).

Characteristics	Pulmonary TB	Extrapulmonary TB	Total
Sex (n)			
Male	46 (38.3%)	17 (14.2%)	63 (52.5%)
Female	33 (27.5%)	24 (20%)	57 (47.5%)
Age (years), Median (IQR)	26 (21–35)	28 (24.5–36)	28 (22–35)
Body weight (Kg), Median (IQR)	53 (45–60)	58 (52.5–68.5)	54.75 (48–61.75)
Drug dose (mg/kg), Mean (95% CI)	4.73 (4.62–4.84)	4.63 (4.47–4.78)	4.7 (4.62–4.78)
Marital status (n)			
Single	54 (45% )	14 (11.67%)	68 (56.7% )
Divorced	2 (1.67% )	1 (0.8%)	3 (2.5%)
Married	22 (18.3%)	24 (20%)	46 (38.3%)
widowed	1 (0.8% )	2 (1.67% )	3 (2.5%)
Educational level (n)			
Illiterate	10 (8.3%)	7 (5.8%)	17 (14.17%)
Primary	29 (24.17%)	13 (10.8%)	42 (35%)
Secondary	27 (22.5%)	16 (13.3%)	42 (35%)
Tertiary	13 (10.8%)	5 (4.17% )	18 (15%)
Smoking (n)			
Yes	15 (12.5%)	1 (0.83%)	16 (13.3%)
No	64 (53.3%)	40 (33.3%)	104 (86.67%)
Khat Chewer (n)			
Yes	19 (15.83%)	2 (1.67%)	21 (17.5%)
No	60 (50%)	39 (32.5%)	99 (82.5%)
Alcohol (n)			
Yes	17 (14.17%)	3 (2.5%)	20 (16.67%)
No	62 (51.67%)	38 (31.67%)	100 (83.3%)

*n* number, *CI* Confidence interval, *IQR i*nterquartile range.

### NAT2 variant allele and genotype frequencies

The frequency distribution of NAT2*4, *5, *6, *7, and *14 alleles in Ethiopian tuberculosis patients were 14.6%, 47.1%, 31.3%, 5.4%, and 1.7%, respectively. There was no significant variation between observed and expected genotype frequencies according to Hardy–Weinberg equilibrium. Genotyping for the four most common functional variant alleles of *NAT2* rs1801280 (c.341 T > C), rs1799930 (c.590G > A), rs1799931 (c.857G > A), and rs1801279 (c.191G > A) was done for all the 120 TB patients enrolled in this study. The four SNP panels reliably estimate acetylator genotype groups^13^. There were twelve *NAT2* genotype groups observed among the study participants. The frequency distribution of the *NAT2* genotype and inferred phenotype is presented in Table 2. The most frequent genotype was NAT2 *5/*5 followed by NAT2 *5/*6, and NAT2 *6/*6. All three were slow acetylators. The frequency of the homozygous wild type (NAT2 *4/*4 genotype) was rare. Among 120 patients enrolled in the study, 4, 27, and 89 patients were fast, intermediate, and slow acetylator genotypes, respectively. The overall frequencies of genotype-predicted slow, intermediate, and fast acetylators were 74.2%, 22.4%, and 3.3% respectively.

**Table 2:** Frequency and percentage distribution of *NAT2* genotype acetylators in Ethiopian tuberculosis patients (N = 120).

*NAT2* genotype	*NAT-2* genotype frequency (n = 120)	*NAT-2* genotype (%)	Acetylator type	Acetylator (%)
NAT-2 *4/*4	4	3.3	Fast	3.3
NAT-2 *4/*14	1	.8	Intermediate	22.4
NAT-2 *4/*5	18	15.0		
NAT-2 *4/*6	7	5.8		
NAT-2 *4/*7	1	.8		
NAT-2 *5/*14	2	1.7	Slow	74.2
NAT-2 *5/*5	29	24.2		
NAT-2 *5/*6	26	21.7		
NAT-2 *5/*7	9	7.5		
NAT-2 *6/*14	1	.8		
NAT-2 *6/*6	19	15.8		
NAT-2 *6/*7	3	2.5		

### Isoniazid plasma exposure

**Figure 1:**
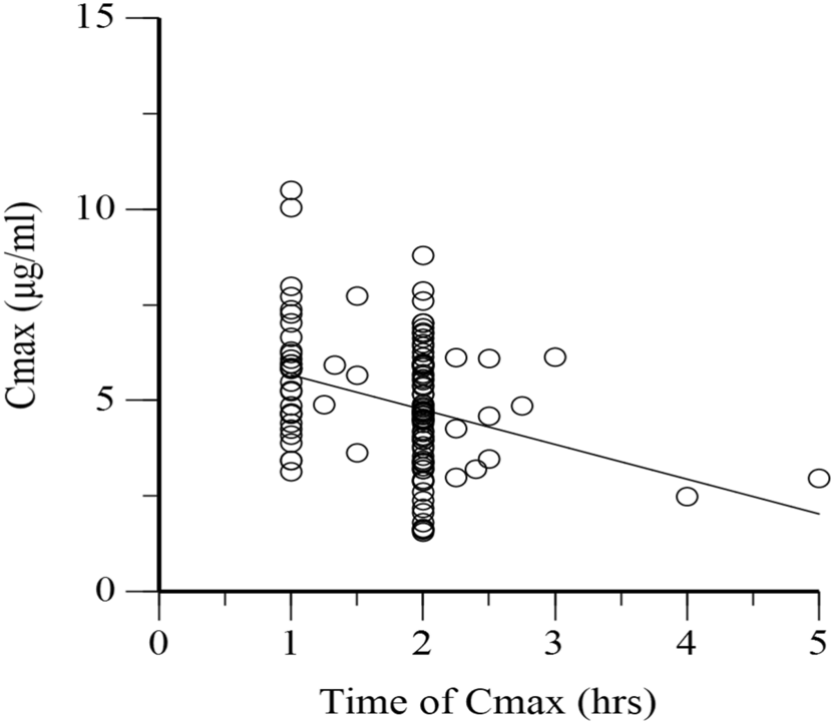
C_max_ of isoniazid compared to the time at which C_max_ achieved.

Spare pharmacokinetic sampling during the intensive phase of the therapy was done (median sampling point = 20 days after anti-TB treatment initiation, range = 11 to 46 days). Plasma sampling took place three times for 112 (92.5%) patients, two times for 7 (5.8%) patients, and one time for 1 (0.8%) patient. Plasma sampling time ranges from 1 to 7 h post-drug intake on an empty stomach. C_max_ was determined by taking the highest of the measured isoniazid plasma concentration. The time at which Cmax was observed is shown in Fig. 1. The regression line in Fig. 1 shows that the highest Cmax is achieved when the plasma is sampled earlier and gradually decreases as the time of sampling increases.

**Figure 2:**
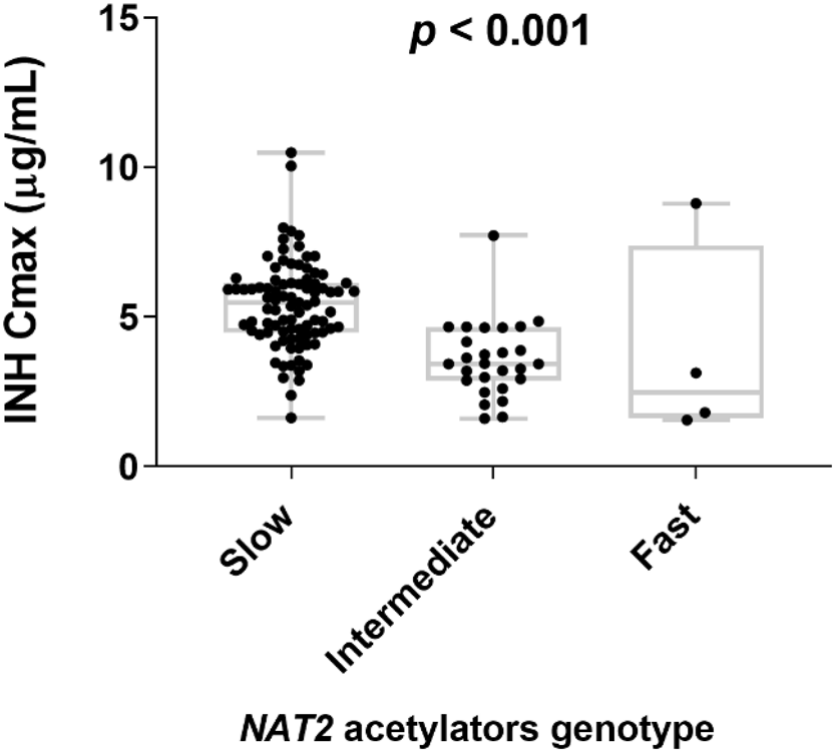
Comparison of isoniazid C_max_ among fast (n = 4), intermediate (n = 27), and slow (n = 89) *NAT2* acetylator genotype groups. The box plots show the median ± interquartile range, while the whiskers denote the minimum and maximum values.

Isoniazid plasma exposure displayed wide between-patient variability, with the median C_max_ being 4.77 µg/mL (IQR 3.78–5.96). A comparison of the median C_max_ of isoniazid (Fig. 2), isoniazid AUC_0-7 h_, and acetyl isoniazid between fast, intermediate, and slow acetylators is shown in Table 3. There was a significant difference in median values of C_max_ of isoniazid and acetyl isoniazid and isoniazid AUC_0–7 h_ among the three *NAT2* acetylators groups. Of the 120 study participants, 15 (12.5%) had an isoniazid C_max_ of < 3 µg/mL (low C_max_), and 28 (23.3%) had a C_max_ of > 6 µg/mL (high) compared to published data. There was no significant difference in isoniazid C_max_ with fast acetylators compared to those with intermediate acetylators (*p* = 0.81). However, the difference in the C_max_ value of isoniazid was significant between fast and slow acetylators (*p* = 0.04) and intermediate and slow acetylators (*p* < 0.001).

**Table 3:** Comparison of isoniazid C_max_ and AUC_0–7 h_, actyl-isoniazid C_max_, and actylisoniazid /isoniazid ratio (Ac-INH/INH) among the three *NAT2* genotypes groups.

Variable		Slow	Intermediate	Fast	*p* value
Isoniazid	C_max_	5.48 (4.49–6.13)	3.43 (2.9–4.4)	2.47 (1.68–5.96)	< 0.001
	AUC_0–7 h_*	13.1 (10.48–15.03)	6.086 (5.21–7.44)	3.73 (2.22–21.87)	< 0.001
Actylisoniazid	C_max_°	0.67 (0.51–0.83)	1.57 (1.1–2.1)	2.21 (1.16–3.44)	< 0.001
Ac-INH/INH		0.12 (0.09–0.167)	0.38 (0.27–0.69)	0.95 (0.3–1.94)	< 0.001

Values are presented as median (interquartile range), AUC_0–7 h -_area under the time-concentration curve, C_max_ -maximum concentration, °data is available for 108 patients, *data is available for 112 patients.

**Figure 3:**
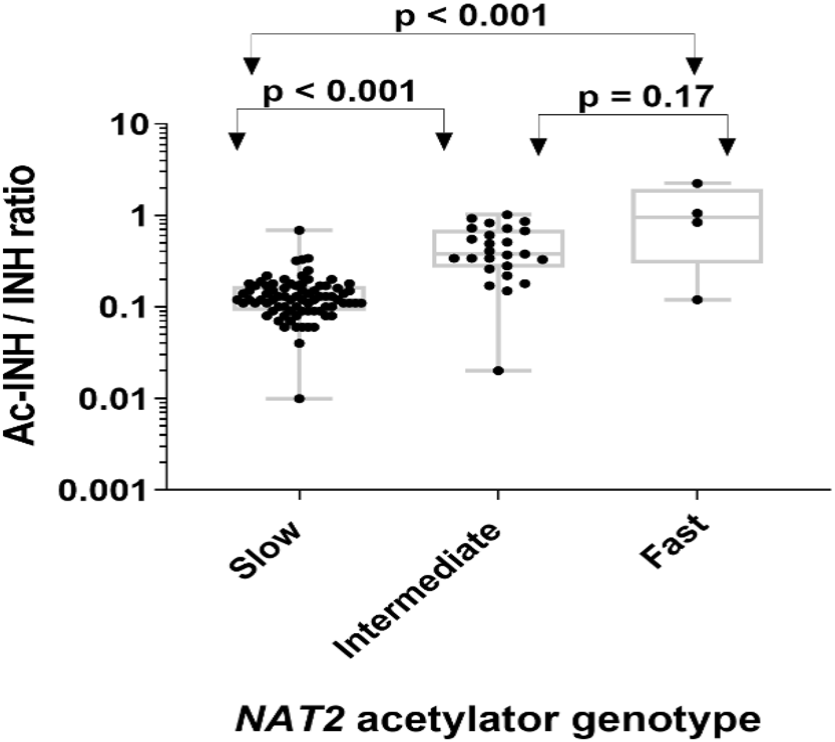
Comparison of acetyl-isoniazid to isoniazid metabolic ratio (Ac-INH/INH) among fast (n = 4), intermediate (n = 25), and slow acetylators (n = 79) genotype groups. The box plots show the median ± interquartile range, while whiskers denote the minimum and maximum values.

There was a significant difference in isoniazid AUC_0–7 h_ between acetylator groups. The overall median isoniazid AUC_0–7 h_ for slow, intermediate, and fast acetylators was 13.09 µg.h/mL, 6.09 µg.h/mL, and 3.73 µg.h/mL, respectively. The variation of AUC_0–7 h_ between the slow genotype group and the other two groups is high (*p* < 0.001). Similarly, acetyl-isoniazid C_max_ concentration varies among the three *NAT2* genotypes. A significant difference in acetyl-isoniazid concentration was observed between slow and intermediate (*p* < 0.001) and slow and fast (*p* = 0.001) acetylators. The difference in AcINH/INH metabolic ratio among the three genotype groups had high variation (*p* < 0.001). A significant difference in AcINH/INH metabolic ratio was observed between slow, intermediate, and slow and fast acetylators. On the other hand, the difference in AcINH/INH metabolic ratio between fast and intermediate metabolizers was statistically non-significant (*p* = 0.17). The pattern of C_max_ and AcINH/INH of the three metabolizer groups is shown in Figs. 2 and 3. At the time of C_max_, ten slow acetylators and two intermediate acetylators had undetectable acetyl isoniazid concentrations. So that the metabolic ratio was available only for 108 patients.

### Predictors of isoniazid plasma exposure

A univariate and stepwise multivariate linear regression model including age, cigarette smoking, khat chewing, alcohol use, gender, drug dose, and the three *NAT2* acetylators genetic variants was done to identify a predictor of isoniazid plasma exposure (Table 4). In the univariate analysis, sex (*p* = 0.001) and *NAT2* acetylator genotypes (*p* < 0.001) were significant variables to predict isoniazid C_max_. In stepwise regression analysis, *NAT2* acetylator genotypes alone, sex and *NAT2* acetylator genotypes, sex, *NAT2* acetylator genotypes, and drug dose (mg) explained 18.9%, 27.6%, and 33% variability in isoniazid C_max_ respectively. Similarly, age, cigarette smoking, khat chewing, and alcohol use, did not predict isoniazid AUC_0–7 h_. Sex (*p* = 0.004), drug dose (mg) (*p* = 0.036), and *NAT2* acetylators genotypes (*p* < 0.001) were significantly associated with variations in isoniazid AUC_0–7 h_. In the multivariate stepwise analysis, Sex, *NAT2* acetylator genotypes and drug dose (mg) were responsible for 35.7% variation in AUC_0–7 h_ of isoniazid. *NAT2* acetylator genotypes alone are responsible for 25.9% of isoniazid AUC_0–7 h_ variation and 29.9% with sex.

**Table 4:** Univariate and multivariate analysis showing factors associated with C_max_ and AUC_0–7 h_ of isoniazid.

PK parameter	Predictor	Univariate analysis		Multivariate analysis	
		Coefficient (β)	*P*	Coefficient (β)	*p*
C_max_	Sex (male vs. female)	1.16	0.001	1.16	< 0.001
	Age	0.01	0.57	–	–
	Smoking (smoker vs. non-smoker)	0.39	0.4	–	–
	Khat (chewer vs. non-chewer)	0.03	0.95	–	–
	Alcohol (user vs. non-user)	0.26	0.53	–	–
	Dose (mg)	0.007	0.066	0.009	0.003
	Acetylator genotypes (fast and intermediate vs. slow)	1.41	< 0.001	1.28	< 0.001
AUC_0–7 h_	Sex (male vs. female)	2.45	0.004	2.36	0.001
	Age	− 0.04	0.32	–	–
	Smoking (smoker vs. non-smoker)	0.05	0.97	–	–
	Khat (chewer vs. non-chewer)	− 0.29	0.8	–	–
	Alcohol (user vs. non-user)	− 0.51	0.66	–	–
	Dose (mg)	0.021	0.036	0.026	0.002
	Acetylator genotypes (fast and intermediate vs. slow)	4.53	0.00	4.26	< 0.001

### Isoniazid metabolic ratio

Plasma acetyl-isoniazid to isoniazid ratio (AcINH/INH) ranged between 0.01 to 2.24 (median = 0.145, IQR = 0.106–0.295). The classification of acetylator phenotypes as slow and fast was done as described by Varshney E et al.^28^ and Aklillu et al.^22^. In brief, a probit plot and regression analysis were used to identify the anti-mode cut-off value to classify slow and rapid acetylators. The cut-off value identified for AcINH/INH ratio was 0.473 and according to this cut-off value**, **86.3% of study participants were classified phenotypically as slow acetylators and the remaining 12.76% as fast acetylators.

### NAT2 genotype—phenotype concordance

The phenotype-genotype concordance was described using traditional phenotype classification. Genotype inferred acetylations status described above. Similarly, using AcINH/INH ratio phenotypic acetylation status as slow and fast acetylators was done as described above. The overall *NAT2* genotype–phenotype concordance was 85%. Concordance between genotype inferred acetylator status and measured NAT2 acetylator phenotype is presented in Table 5. *NAT 2* genotype predicted acetylator phenotype in 92 patients accurately. Almost all slow acetylator genotypes (98.3%) were accurately predicted, whereas 13.88% of fast acetylators genotypes were predicted as slow acetylator phenotypes. Only one NAT2*5/*5 slow acetylator genotype was predicted as a fast acetylator phenotypically. Heterogeneity was observed for NAT2*4/*5 and *4/*6 on the acetylation status. More than half of NAT2*4/*5 (62.5%) and NAT2*4/*6 (57%) genotype carriers were slow acetylator phenotypically.

**Table 5:** Concordance between genotype inferred acetylator status and measured NAT2 acetylator phenotypes in Ethiopian tuberculosis patients (χ2 test *p* < 0.001).

Genotype inferred *NAT 2* acetylator status	Phenotype inferred NAT 2 acetylator status (AcINH to INH ratio)		
	Fast	Slow	Total
Rapid	3 (21.4%)	1 (1.1%)	4 (3.7%)
Intermediate	11 (78.5%)	14 (15%)	25 (23.15%)
Slow	1 (7.1%)	78 (83.87%)	79 (73.15%)
Total	15 (12.96%)	93 (86.11%)	108

## Discussion

The effect of *NAT2* genotype on the pharmacokinetics of isoniazid in TB patients is well explored in various Asian and Caucasian populations but data is scarce from sub-Sharan Africa, including Ethiopia, the seventh top high-TB burden country globally and the 2nd most populous nation in Africa. Ethiopians display wide pharmacogenetics variations compared to other populations within and outside of Africa^29,30^. In this study, we investigated the profile and predictors of isoniazid plasma exposure and the effect of the *NAT2* genotype on isoniazid and its metabolite acetyl isoniazid pharmacokinetics in a cohort of newly diagnosed Ethiopian tuberculosis patients. Our main findings include i) a significant association of *NAT2* acetylator genotype with between-patient variability in isoniazid pharmacokinetics (C_max_, AUC_0–7 h_, metabolic ratio), ii) a high concordance rate (85%) between *NAT2* genotype and acetylation rate of isoniazid, iii) high prevalence of slow acetylators in Ethiopian TB patients and the majority of (85%) achieved therapeutic isoniazid plasma concentration. iv) *NAT2* genotype and sex are significant predictors of isoniazid plasma exposure.

Interestingly, we found a high prevalence of genotypic (74.2%) and phenotypic (86%) slow acetylators in Ethiopian TB patients. Genotypically, 22.4% were intermediate acetylators, and only 3.3% were fast acetylators. Our finding is in line with a previous study among healthy Ethiopians, reporting the frequency of slow, intermediate, and fast acetylators being 73.6%, 24.6%, and 1.8%, respectively^22^. The frequency distribution of the slow acetylators genotype varies between populations. About 10–20% of Asians and 40–70% of Caucasians are slow acetylators^31^. Black Africans, the most genetically diverse population on earth, display the highest level of within‐population diversity of *NAT2* genotype and outside of the region^19,20^. The fast acetylators are predominant in West Africa. Compared to the Ethiopians, a lower frequency of slow acetylators in Senegalese (44.3%)^32^, South African (52.5%)^33^, and Tanzanians (48%) TB patients^34^ is reported. This confirms the wide heterogeneity of black Africans and results from one population may not apply to others within the region.

Various levels of concordance between the *NAT2* genotype and acetylation phenotype are reported. Our study revealed high concordance (85%) between *NAT 2* genotype and NAT2 acetylation phenotype. Aklillu et.al^22^ reported a lower (75%) but significant *NAT2* genotype–phenotype concordance in healthy Ethiopians using caffeine as a probe drug for NAT2 enzyme activity. Unlike our finding in Ethiopian TB patients, a recent study in Zulu-speaking South Africans reported a lower percentage (55%) of slow acetylators and poor or no significant concordance between the *NAT2* genotype and isoniazid phenotype concordance^33^.

Low isoniazid concentrations have been postulated to result in unfavorable treatment outcomes^35,36^. A target of 3–6 μg/mL for the peak concentration (C_max_) following a 300-mg daily dose of isoniazid is considered vital for a favorable treatment outcome^35^. Studies also reported that anti-TB drug-induced hepatotoxicity was associated with slow acetylation^37^. In this study, the C_max_ of isoniazid was greater than 3 µg/mL in 87.5% of patients and the AUC_0–7 h_ of isoniazid was high suggesting high isoniazid exposure in Ethiopian tuberculosis patients. A similar pattern of isoniazid plasma concentration was observed in Ethiopian pediatric TB patients^21^. The large proportion of slow acetylators in our cohort means isoniazid plasma exposure is sufficiently high to provide clinical benefit.

In univariate and multivariate analysis, sex, and *NAT2* acetylator genotype status were predictors of isoniazid C_max_. Females had higher C_max_ compared to males, which is in agreement with those of previous studies^38,39^. This may explain the previous finding of an increased risk of isoniazid-induced drug toxicity^40^ and a lower risk of unfavorable treatment outcomes in females^41^. Plasma isoniazid C_max_ increased as the isoniazid dose increased. This suggests that dose is also a predictor of C_max_. Nonsmokers had higher isoniazid C_max_ than smokers; inversely khat chewer had higher isoniazid AUC_0–7 h_ than nonchewers though the differences in both were not significant.

NAT2 enzyme activity is the rate-limiting step in acetylating isoniazid to acetyl isoniazid. A high interindividual variation was observed in the clinical efficacy, elimination, and side effects of isoniazid. These variations were related to the difference in the NAT2 enzyme which metabolizes isoniazid. We observed a bimodal isoniazid C_max_, unlike other studies which reported trimodal C_max_ and AUC based on *NAT2* genotype^42,43^. A significant variation of isoniazid C_max_ and AUC_0–7_ were observed between the slow and the other two acetylators groups while there is no significant variation between fast and intermediate acetylators. The low number of fast acetylators in our study population might be attributed to the absence of difference between fast and intermediate acetylators groups. Fast isoniazid acetylators showed lower C_max_ and exposure to the drug than slow acetylators. Several authors reported an increased risk of toxicity in slow acetylators^37,44^ and an increased risk of therapeutic failure in fast acetylators^34,45^ patients. *NAT2* genotype-guided isoniazid administration reduced toxicity and improved treatment outcomes in Japanese trials^46^. Thus, owing to high exposure to isoniazid, Ethiopians are at increased risk of toxicity from isoniazid. Indeed high rates of anti-TB and antiretroviral treatment-induced liver toxicity in Ethiopian TB-HIV coinfected patients, particularly in slow acetylators is reported previously^47^.

We evaluated the plasma isoniazid C_max_ and drug exposure following standard laboratory recommendations like collecting plasma from patients who have received anti-drug after fasting overnight. Several studies reported that food decreased absolute bioavailability and maximum concentration of isoniazid^48,49^. Plasma was immediately separated and kept at –20 °C until transported for storage at − 80 °C on the same day. The cold chain was kept during sample transportation. The concentration of isoniazid after a week of storage at -20 °C was about 80% of the initial amount and no significant change in the initial concentration was observed if stored at -80 °C for longer than six months^50^. Study participants were patients receiving a standard dose of isoniazid in a fixed dose combination with rifampicin, ethambutol, and pyrazinamide. Patients had no prior exposure to the drugs and had no reported comorbidities of liver, kidney, HIV infection, or diabetes. Low isoniazid concentration was observed in TB-HIV co-infected patients^51^.

Our study has some limitations. Although the spare sampling strategy is evolving in recent years and found to be useful to capture AUC0-24 h^52^, the time point at which we collected the plasma sample varied from patient to patient. The sparse sampling strategy may not fully define the individual C_max_ and AUC. We enrolled 120 patients in both the pharmacokinetics and pharmacogenetics studies. Because of the low frequency of fast acetylators in our study participants, we did not observe significant pharmacokinetics variation between fast and intermediate acetylators.

In conclusion, we report a high prevalence of the slow *NAT2* acetylator genotype in Ethiopian tuberculosis patients. NAT2 acetylation status and the female sex are strong predictors of isoniazid plasma concentrations. The majority of the patients attain therapeutic plasma isoniazid exposure for a favorable treatment outcome. On the other hand, slow acetylators and females are at a higher risk of concentration-dependent isoniazid toxicity. Therefore, close safety monitoring, particularly for patients on high-dose isoniazid short-course MDR-TB therapy is recommended for early identification and management of treatment-associated adverse events.

## Data Availability

All data generated or analyzed in this study are included in this article. The datasets used and/or analyzed during the study are available from the corresponding author upon reasonable request.

## References

[1] WHO, Global tuberculosis report 2022. Geneva: World health organization; 2022. Licence: CC BY-NC-SA 3.0 IGO. (2022).

[2] Sandhu GK (2011). Tuberculosis: Current situation, challenges and overview of its control programs in India. J. Glob. Infect. Diseas..

[3] Pontali E, Raviglione MC, Migliori GB (2019). Regimens to treat multidrug-resistant tuberculosis: Past, present and future perspectives. Eur. Respir. Rev..

[4] Saravanan M (2018). Review on emergence of drug-resistant tuberculosis (MDR & XDR-TB) and its molecular diagnosis in Ethiopia. Microb. Pathog..

[5] Chigutsa E (2015). Impact of nonlinear interactions of pharmacokinetics and MICs on sputum bacillary kill rates as a marker of sterilizing effect in tuberculosis. Antimicrob. Agents Chemother..

[6] Pasipanodya JG, Srivastava S, Gumbo T (2012). Meta-analysis of clinical studies supports the pharmacokinetic variability hypothesis for acquired drug resistance and failure of antituberculosis therapy. Clin. Infect. Dis..

[7] Murray JF, Schraufnagel DE, Hopewell PC (2015). Treatment of tuberculosis. A historical perspective. Ann. Am. Thorac. Soc..

[8] Dooley KE (2020). Early bactericidal activity of different isoniazid doses for drug-resistant tuberculosis (INHindsight): A randomized, open-label clinical trial. Am. J. Respir. Crit. Care Med..

[9] Donald PR (1997). The early bactericidal activity of isoniazid related to its dose size in pulmonary tuberculosis. Am. J. Respir. Crit. Care Med..

[10] Peloquin CA (2002). Therapeutic drug monitoring in the treatment of tuberculosis. Drugs.

[11] Kinzig-Schippers M (2005). Should we use N-acetyltransferase type 2 genotyping to personalize isoniazid doses?. Antimicrob. Agents Chemother..

[12] Sohni YR (2001). Active electronic arrays for genotyping of NAT2 polymorphisms. Clin. Chem..

[13] Hein DW, Doll MA (2012). Accuracy of various human NAT2 SNP genotyping panels to infer rapid, intermediate and slow acetylator phenotypes. Pharmacogenomics.

[14] Headriawan A (2021). NAT2 gene rs1041983 is associated with anti-tuberculosis drug induced hepatotoxicity among pediatric tuberculosis in bandung. Indonesia. Appl. Clin. Genet..

[15] Ben Mahmoud L (2012). Polymorphism of the N-acetyltransferase 2 gene as a susceptibility risk factor for antituberculosis drug-induced hepatotoxicity in Tunisian patients with tuberculosis. Pathol. Biol. (Paris).

[16] Wang P (2016). Isoniazid metabolism and hepatotoxicity. Acta Pharm. Sin. B.

[17] Zhang M (2018). The association between the NAT2 genetic polymorphisms and risk of DILI during anti-TB treatment: A systematic review and meta-analysis. Br J. Clin. Pharmacol..

[18] Wang P (2016). Isoniazid metabolism and hepatotoxicity. Acta Pharmaceutica Sinica B.

[19] Podgorná E (2015). Variation in NAT2 acetylation phenotypes is associated with differences in food-producing subsistence modes and ecoregions in Africa. BMC Evol. Biol..

[20] Mortensen HM (2011). Characterization of genetic variation and natural selection at the arylamine N-acetyltransferase genes in global human populations. Pharmacogenomics.

[21] Misgana Ibrahim1, Ephrem Engidawork1*, Getnet Yimer3, Kidist Bobosha2 and A. Aseffa2, Pharmacokinetics of isoniazid in Ethiopian children with tuberculosis in relation to the N-acetyltransferase 2 (NAT2) genotype. Vol. Vol. 7. 2013: African Journal of Pharmacy and Pharmacology.

[22] Aklillu E (2018). N-acetyltransferase-2 (NAT2) phenotype is influenced by genotype-environment interaction in Ethiopians. Eur. J. Clin. Pharmacol..

[23] Yimer G (2008). Anti-tuberculosis therapy-induced hepatotoxicity among ethiopian HIV-positive and negative patients. PLoS ONE.

[24] O. , World Health (2021). WHO global lists of high burden countries for tuberculosis (TB), TB/HIV and multidrug/rifampicin-resistant TB (MDR/RR-TB), 2021–2025: Background document.

[25] health-Ethiopia, M.o., Guidelines for clinical and programmatic management of TB, TB/HIV, DR-TB and leprosy in ethiopia. (2021).

[26] McDonagh EM (2014). PharmGKB summary: Very important pharmacogene information for N-acetyltransferase 2. Pharmacogenet. Genomics..

[27] Agency, E.M., Guideline-bioanalytical-method-validation. (2011). London United Kingdom.

[28] Varshney E (2012). Prevalence of poor and rapid metabolizers of drugs metabolized by CYP2B6 in North Indian population residing in Indian national capital territory. Springerplus.

[29] Mugusi, S., et al., Impact of population and pharmacogenetics variations on efavirenz pharmacokinetics and immunologic outcomes during anti-tuberculosis co-therapy: a parallel prospective cohort study in two sub-sahara african populations. Front. Pharmacol., (2020). **11**. 10.3389/fphar.2020.00026 PMC7019112 32116703

[30] Aklillu E (2014). High CYP2A6 enzyme activity as measured by a caffeine test and unique distribution of CYP2A6 variant alleles in Ethiopian population. OMICS.

[31] Djordjevic N (2011). N-Acetyltransferase-2 (NAT2) gene polymorphisms and enzyme activity in Serbs: Unprecedented high prevalence of rapid acetylators in a white population. J. Clin. Pharmacol..

[32] Toure A (2016). Prevention of isoniazid toxicity by NAT2 genotyping in Senegalese tuberculosis patients. Toxicol. Rep..

[33] Mthiyane, T., et al., N-acetyltransferase 2 genotypes among zulu-speaking south Africans and Isoniazid and N-acetyl-isoniazid pharmacokinetics during antituberculosis treatment. Antimicrob Agents Chemother, 2020. **64**(4). 10.1128/AAC.02376-19 PMC7179278 31964788

[34] Denti P (2015). Pharmacokinetics of isoniazid, pyrazinamide, and ethambutol in newly diagnosed pulmonary TB patients in tanzania. PLoS ONE.

[35] Alsultan A, Peloquin CA (2014). Therapeutic drug monitoring in the treatment of tuberculosis: An update. Drugs.

[36] Sileshi T (2021). The impact of first-line anti-tubercular drugs' pharmacokinetics on treatment outcome: A systematic review. Clin. Pharmacol..

[37] Wattanapokayakit S (2016). NAT2 slow acetylator associated with anti-tuberculosis drug-induced liver injury in Thai patients. Int. J. Tuberc. Lung. Dis..

[38] McIlleron H (2012). Reduced antituberculosis drug concentrations in HIV-infected patients who are men or have low weight: Implications for international dosing guidelines. Antimicrob. Agents Chemother..

[39] Deshmukh, S., et al., Sex differences in tb clinical presentation, drug exposure, and treatment outcomes in India. Chest, (2022). 10.1016/j.chest.2022.09.024 PMC10258435 36174745

[40] Pettit AC (2013). Female sex and discontinuation of isoniazid due to adverse effects during the treatment of latent tuberculosis. J. Infect..

[41] Deshmukh, S., et al., Sex differences in tuberculosis clinical presentation, drug exposure, and treatment outcomes in India. Chest, (2022). 10.1016/j.chest.2022.09.024 PMC10258435 36174745

[42] Parkin DP (1997). Trimodality of isoniazid elimination: Phenotype and genotype in patients with tuberculosis. Am. J. Respir. Crit. Care Med..

[43] Wilkins JJ (2011). Variability in the population pharmacokinetics of isoniazid in South African tuberculosis patients. Br. J. Clin. Pharmacol..

[44] Mushiroda T (2016). Development of a prediction system for anti-tuberculosis drug-induced liver injury in Japanese patients. Hum. Genome Var..

[45] Jung JA (2015). A proposal for an individualized pharmacogenetic-guided isoniazid dosage regimen for patients with tuberculosis. Drug. Des. Devel. Ther..

[46] Azuma J (2013). NAT2 genotype guided regimen reduces isoniazid-induced liver injury and early treatment failure in the 6-month four-drug standard treatment of tuberculosis: A randomized controlled trial for pharmacogenetics-based therapy. Eur. J. Clin. Pharmacol.

[47] Yimer G (2011). Pharmacogenetic & pharmacokinetic biomarker for efavirenz based ARV and rifampicin based anti-TB drug induced liver injury in TB-HIV infected patients. PLoS ONE.

[48] Kumar AKH (2017). Food significantly reduces plasma concentrations of first-line anti-tuberculosis drugs. Indian J. Med. Res..

[49] Requena-Méndez A (2018). Intra-individual effects of food upon the pharmacokinetics of rifampicin and isoniazid. J. Antimicrob. Chemother..

[50] Tron, C., et al., Stability study of isoniazid in human plasma: practical aspects for laboratories. Therapeutic. Drug. Monitoring, **37**(6), (2015). 10.1097/FTD.0000000000000218 25945417

[51] Wiltshire CS (2014). Low isoniazid and rifampicin concentrations in TB/HIV co-infected patients in Uganda. J. Int. AIDS Soc..

[52] Cojutti P (2017). Limited sampling strategies for determining the area under the plasma concentration-time curve for isoniazid might be a valuable approach for optimizing treatment in adult patients with tuberculosis. Int. J. Antimicrob. Agents.

